# Short-Time Fourier Transform Based on Metaprogramming and the Stockham Optimization Method

**DOI:** 10.3390/s21124123

**Published:** 2021-06-15

**Authors:** Grzegorz Rybak, Krzysztof Strzecha

**Affiliations:** Institute of Applied Computer Science, Lodz University of Technology, 90-537 Lodz, Poland

**Keywords:** STFT, program optimization, butterfly operation, Stockham algorithm, metaprogramming, Java, DSP, ECT

## Abstract

The extension for high-performance STFT (Short-Time Fourier Transform) algorithm written entirely in Java language for non-parallel computations is presented in the current paper. This solution could compete with the best available and most common algorithms supplied by libraries such as FFTW or JTransform. The main idea was to move complex computations and expensive functions to the program generation phase. Thus, only core and essential operations were executed during the runtime phase. Furthermore, new approach allows to eliminate the necessity for a rearrangement operation that uses the bit-reversal permutation technique. This article presents a brief description of the STFT solution that was worked out as an extension for the original application, in order to increase its efficiency. The solution remains a Stockham algorithm adapted using metaprogramming techniques and entails an additional reduction its execution time. Performance tests and experiments were conducted using a Java Platform and JMH library, which allowed for accurate execution time measurements. Major aspects of the Java VM like warm-up effects were also taken into consideration. Solution was applied into Electrical Capacitance Tomography measurement system in order to measure the material changes during the silo discharging industrial process.

## 1. Introduction

During silos discharging process, particularly with grain or other loose material, dynamic effects frequently occur. The dynamic effects and other related to material conveying processes phenomena have a major impact to the surrounding environment leading to material depreciation or even industrial disasters. The silo vibrations are commonly caused or induced by material discharge and a resonance between the eigen frequency of the silo structure, the frequencies of self-excited material particles and the frequencies of the operating machines [1].

One of the process tomography technique is Electrical Capacitance Tomography (ECT). This allows to measure parameters, such as distribution of the material concentration in the cross section of the silos, the change of material concentration in time during filling and emptying the silo and the vibrations of the transported material. STFT algorithm presented in this article was used to measure such frequency phenomena [2].

The methods for signal analysis like Frequency transform are still an essential tool in digital signal processing (DSP). They are used by many algorithms, e.g., linear filtration, correlation and frequency spectrum analysis. Particularly Discrete Fourier Transform (DFT) and its optimized version Fast Fourier Transform (FFT) are used. Both give good results for signals from stationary systems, but are not sufficient to describe signals generated by systems whose properties change over time (not stationary). Such signals require special treatment using dedicated analysis techniques. One such technique is the Short-Time Fourier Transform (STFT), a very popular tool used in analysis of frequency changes characteristic in time domain that provides information of harmonics magnitude in consecutive time ranges called time windows [3]. This method is also called the Time-Varying Fourier Transform (TVFT) [4]. There are also other techniques as wavelet transform [5], but not discussed in this paper.

The STFT is a key component of signal processing systems in many areas such as medicine, industry measurement and control, audio signals analysis.

Many implementations of the STFT algorithm have been proposed, which differ in terms of their memory and time complexity. Straightforward implementations of STFT are not used, as they may have very high computational cost. The STFT may be computed with filter banks or as consecutive FFT operations. It is possible to obtain transforms in an iterative manner or using a recursive approach. Both ways have advantages and disadvantages. The second approach requires additional memory for functions invocation stack but the solution is more readable. When algorithm does not require more memory for data processing than input array, it is called in-situ or “in place” solution [6]. On the other hand, additional memory may allow to increase the performance.

The Sliding Window Discrete Fourier Transform SDFT is also known. Despite giving incredible results where time complexity equals O(*n*), SDFT is associated with error propagation, as each subsequent result is based on the previous one [7].

Beside this, wide variety of optimization methods was introduced and described [8]. The basic ones are the decimation in time (the time samples are rearranged in alternating groups) and the decimation in frequency (the frequency samples are computed separately in alternating groups) [6]. In both cases a different radix may be used. Most often is: radix-2 or radix-4. Also mixed-radix and split-radix approaches are used. Great diversity exists in the techniques for ordering and accessing the data at each stage of the FFT computation. Optimizations in bit-reversal permutation are used. The Stockham auto-sort algorithm that uses the properties of the SIMD (Single Instruction, Multiple Data) architecture should be mentioned here. Although some digital signal processors (DSPs) provide native bit reversal operations (hardware solutions), many modern desktop CPUs, do not have an opcode to perform such function [9]. Another problem is developing an in-place algorithm that overwrites its input with output with O(1) memory complexity. Popular optimization tricks such as look-up tables roots of unity are also commonly used. There are also optimizations designed for hardware implementations such as feed forward STFT.

Research conducted in the last decade on improving the performance of the Fourier transform focuses primarily on the use of various parallel hardware architectures [10,11]. Particular attention should be paid to implementations that use modern graphics processors [9,12,13,14]. Such solutions have been shown to give good results, both in terms of efficiency and precision. NVIDIA, a leading manufacturer of graphics processors, Nvidia offers a highly optimized cuFFT library that enables FFT calculations on its processors [15]. Solutions that use other optimization techniques apart from graphics processors are also proposed [16,17]. It is also worth mentioning the works on the implementation of FFT in programmable FPGA structures [18,19]. All of the above optimization techniques also apply to STFT. The use of GPUs to calculate STFT has been described among others in [20,21,22]. Attempts to implement STFT in FPGA structures are presented, for example, in [23,24].

A separate group of optimization methods, not yet fully recognized, are metaprogramming techniques. Especially in the relation to STFT, the metaprogramming has proven to be useful [8]. Previous research, particularly realizations in C++ using functions templates, has shown the benefits of this approach for FFT [25,26].

Implementing complex algorithms such as STFT usually requires finding a reasonable compromise between execution time and memory requirements. Resource limitations have an unquestionable impact on the techniques that may be used. Storage issues are not as crucial as they were a few years ago, but execution time still remains an issue. Thus, any improvement to common algorithms increases their usability.

Until recently, metaprogramming was considered a relatively new technique. Nowadays we can see a large number of studies on this issue, which is still being developed [26,27,28,29]. Metaprogramming refers to a group of generative programming techniques [28]. It can be explained as programming at a higher level of abstraction, using another program (metaprogram) to obtain the target application [29]. Metaprogramming may thus be described as an automatic approach to program creation [30].

Metaprogramming techniques can be divided into three groups:

- aspect programming paradigm (AOP) [27],
- dynamic metaprogramming,
- static metaprogramming.

AOP refers to code injections in an implicit manner [1]. The external part of the script is applied to the target application in places that have been earlier specified. Adding aspect code to the program can take place before its launch as well as during operation. Aspect programming is often used for code optimization and generalization.

Dynamic metaprogramming covers techniques such as introspection, reflection, dynamic polymorphism, and interpreters.

Static metaprogramming allows for precise code preparation before runtime, during which expensive computations are transferred outside. Static metaprogramming covers multiple solutions, such as preprocessors, compilators, linkers, macro, code templates, inline functions, and other code generators. The optimization techniques involve: unwinding the loop (loop body code multiplication that leads to reduction of expensive ‘if’ or ‘jump’ instruction usage); vectorization [31] (additional SIMD operations utilizations and low-level instruction execution during runtime); parametrization (the ability to generate final code based on its domain parameters); code templates (as part of static polymorphism); loop tiling or lookup table preparation [8].

Although metaprogramming techniques have been known for years, there has been little attempt to apply them to digital signal processing algorithms so far. Possibilities of using metaprogramming in DSP are discussed in [32]. An example implementation of Feedback Delay Network (FDN) artificial reverberation algorithm is also presented there. In [33] a Python non-uniform fast Fourier transform (PyNUFFT) package has been presented. Metaprogramming libraries have been employed for its acceleration. GPU implementation of FFT and DFT using meta programming optimization techniques is presented in [34].

In particular, a group of static metaprogramming techniques is particularly interesting for the purpose of optimizing the STFT. STFT algorithm has a large number of intermediate products obtained during code execution that can be successfully moved into the code generation phase. Obviously, this is extremely costly in terms of memory, but at the same time it derives a significant profit in terms of execution time.

The research presented below in the article was aimed at reducing the computational complexity of the STFT calculation algorithm with the use of various optimization techniques, with particular emphasis on metaprogramming implementation techniques.

## 2. Method

In order to increase STFT algorithm performance many optimization methods are used:

- decimation in time or frequency (divide and conquer mechanism),
- radix-4 decimation optimization (omits part of complex computation),
- Euler transform,
- lookup tables for root of unity,
- bit-reversal permutation optimizations (for an instance a SIMD optimizations),
- feedforward techniques,
- in-situ approach (low memory requirements),
- parallelism,
- group of metaprogramming techniques (loop unwinding, vectorization, parametrization, code templates, arithmetic operation reduction by moving part of them to metaprogram).

The Danielson-Lanczos lemma enables the Fourier Transform to be computed in a faster way than using a naive DFT algorithm [6,35]. In this approach, the input sequence is divided into two subsequences, a consecutive series of odd and even indices. Such decomposition is called decimation, in this case decimation in time (DIT). There is also decimation in frequency (DIF), and both methods require *N*/2*log_2_(*N*) complex additions, subtractions and multiplications. When the decimation base is equal to 2 it is called RADIX-2 decimation. This has an additional impact on input data. The input series should have the size of the power of two, to divide the sequence into two subseries of equal length in recursive stages. The division ends when there are only one-element arrays.

In commonly-used applications, there are several techniques for time series division. For instance, RADIX-4 or the more efficient Split-RADIX may be used. When the input array has *N* elements, and *N* is not a prime number, factorization is possible, whereby, for example, from a 15-element array we obtain 3 arrays consisting of 5 elements or 5 arrays with 3 elements.

Both computations, DIT and DIF can be performed in-situ what means that it doesn’t require additional memory. The disadvantage is that the output is not in order. It is possible to obtain output in order, but the computation cannot be done in-situ, and at least one additional array must be provided [6].

**Figure 1:**
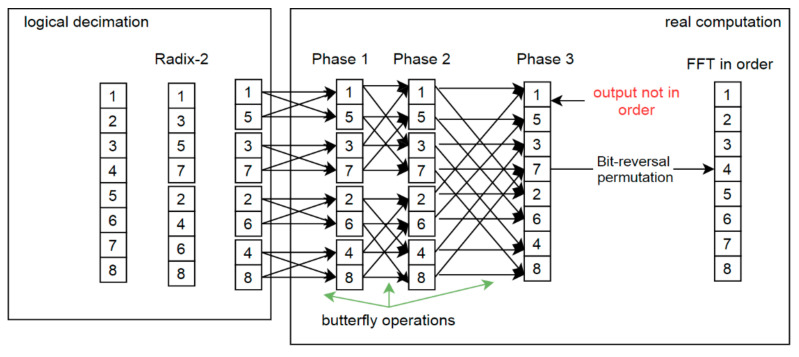
Fast Fourier Transform and bit-reversal permutation; indices division on the left; merge phase on the right side; final computation requires reordering to obtain expected output.

The FFT computation process is presented in Figure 1. On the left-hand side there is RADIX-2 decimation. This is only a logic decomposition to present the idea of the second step of real computations. Then two-element sequences are combined (DFT). For real numbers, such operations are trivial and quite fast. There are no complex number multiplications. The next stages require complex number arithmetic, which uses so-called twiddle factors whereby values depend on the subsequence length and the position of the current computation pair. Twiddle factors are often obtained from a look-up table, as one memorization technique.

In metaprogramming, these values also may be explicitly placed in execution code, significantly increasing its performance. Each stage returns a partial output that is used in the following stage. The number of stages depends on the length of the input series. When RADIX-2 decimation is used, there are log_2_(*n*) stages, so for example in Figure 1 the given array is 8 in size and there are 3 stages. Finally, after all processing stages there is a Fourier Transform output with *N* complex numbers but disordered (Figure 1).

To obtain an output in the appropriate order, the indexes should be rearranged. Here, several solutions are allowed. One of them is a bit-reversal permutation [6], which is applied with time-execution complexity O(*n*). In the example presented below (Equation (1)), which describes a situation where the possible values are in the range of [0, 7] giving k = 3 digits in binary system, it can be seen that the values 0_10_ and (2*^k^*−1)_10_ are unchanged.

K = 0_10_ = 000_b_ and k = 3 => bit_rev_permutation(K) = 000_2_ = 0_10_ ← unchanged K = 6_10_ = 110_b_ and k = 3 => bit_rev_permutation(K) = 011_2_ = 3 _10_ K = 7_10_ = 111_b_ and k = 3 => bit_rev_permutation(K) = 111_2_ = 7_10_ ← unchanged (1)

The number of array operations *A_op_* is lower than n (*A_op_* < *n*). Nonetheless, it is possible to omit this expensive overhead, as Swarztrauber observed [36]. Basically, each of the algorithms presented in Swarztrauber’s paper computes the same result. They differ only in the ways that the intermediate computations are stored. These solutions present the possibility of adapting FFT mainly to vector computers. Cooley-Tukey and Pease algorithms compute the FFT in a permuted order, thus often a separate order phase is used to rearrange the output elements. This operation is time-consuming not only on vector computers.

In a paper that is fundamental to the early FFT literature [6] W.T. Cochran introduced an autosort FFT algorithm and attributed it to Stockham. The Stockham autosort algorithm has received much attention, because it computes the Fourier Transform in the proper order without an explicit order phase. Despite the fact that solution requires additional memory, bit-reversed permutations has very expensive arithmetic and read/write overhead operation, thus it is reasonable to use this method. Listing 1 shows Stockham solution written in FORTRAN [36]. To omit the sorting phase, an additional table is used that allows the data to be kept in order.

**Listing 1:** The code of Stockham solution for FFT computation. Red color presents auxiliary array adjustment for reordering purposes and green color indicates two procedures that were used (code from [36]).

SUBRUTINE **STOCK**(IS, M, C, WORK) C THE STOCKHAM AUTOSORT FFT COMPLEX C(1), WORK(1) N = 2 ** M C Done for each FFT stage—depends on log; N DO 100 L = 1,M LS = 2 ** (L-1) NS = N/(LS + LS) CALL STOCK1 (IS, LS, NS, C,**WORK**) DO 100 I = 1, N C(I) = **WORK**(I) 100 CONTINUE RETURN END SUBRUTINE **STOCK1**(IS, LS, NS, C, **CH**) COMPLEX OMEGA, WYK, C(NS,2, LS), CH(NS, LS,2) ANGLE = FLOAT(IS) * 4. * ATAN(1.)/FLOAT(LS) OMEGA = CMPLX(COS(ANGLE), SIN(ANGLE) DO 200 J= 1,NS WYK = OMEGK * C(J, 2, I) **CH**(J,I,1) = C(J,1, I) + WYK **CH**(J,I,2) = C(J,1,I) − WYK OMEGK = OMEGA * OMEGK 200 CONTINUE RETURN END

**Figure 2:**
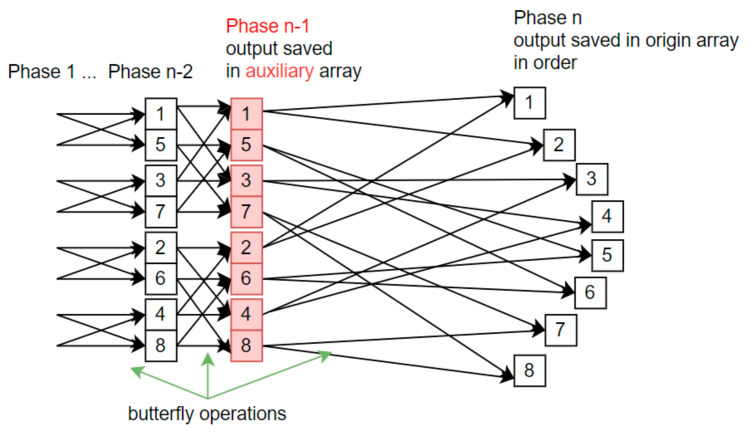
Fast Fourier Transform with Stockham algorithm adaptation; auxiliary array (red) used for implicit reordering.

Algorithms presented on Figure 1 and Figure 2 inspired the concept of applying the Stockham solution to metaprogram the STFT algorithm. There was no need to keep additional table for whole execution time. In fact, it does not matter how the computations are performed during first stages. Only the last stage and its final output is important. In what follows, STFT modifications are presented that keep output data in order without requiring additional arithmetic and input/output operations. Depending on the processing stage, the initial or auxiliary array is used, as shown in Figure 2.

While the consecutive stages (up to phase *n*-2) are executed, the solution remains in-situ. For phase *n*-1, the result is saved into an auxiliary array with a length of *N*-complex. The calculations themselves remain unchanged. In the final phase, *n* computations are performed with data from an aux array and the output is stored in the initial array in the correct order. To obtain this behavior, the addressing mechanism is changed and bit-reversal permutation coefficients are used only in the metaprogram. There are no reordering operations in run-time, so its execution time is reduced.

**Figure 3:**
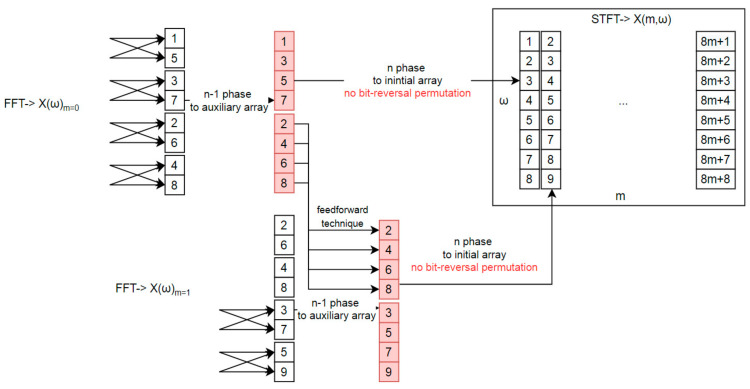
Short-time Fourier Transform with Stockham algorithm adaptation; figure shows auxiliary array adaptation for feedforward technique; data processing between successive FFT frames.

Figure 3 shows a Short-Time Fourier Transform based on the above solution. In this example, the first two time windows are presented: *m* = 0, *m* = 1. The previous stages have been calculated as for regular FFT. The output of stage *n*-1 for the first FFT (*m* = 0) is written into an additional array that was highlighted with red color on Figure 3. The output from the last stage (*n*) is moved back to the initial array in the appropriate order.

**Figure 4:**
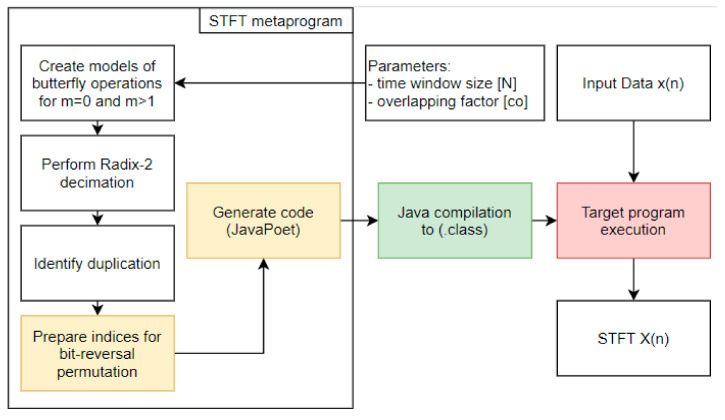
Short-time Fourier Transform with metaprogramming technique block diagram; metaprogram on the left; STFT runtime phase on the right side. Solution without Stockham optimization.

It is important to note that a feed-forward technique was applied to the metaprogramming solution. This reduces the number of computations, due to the fact that duplicated butterfly operations exist in the process [37]. This can have a major impact on the application. If we introduce an additional array, the data moved from the previous time window (*m*−1) should be copied to both the initial and auxiliary arrays, depending on the indices and the STFT overlapping factor. Figure 4 presents a rectangular time window and an overlapping factor equal to 1, which means that the time window is moved over the input data one by one sample. Thanks to this approach, there is no need to perform bit-reversal permutation rearrangement.

Implementation was performed using the Java Platform with the JavaPoet library (JavaPoet; https://github.com/square/javapoet; accessed on 10 January 2021; author: Jake Wharton, version: 1.10.0). This enabled a large part of the algorithm to be moved into the metaprogram, such as indices management in loops, arithmetic and logical operations that do not depend on input data but only on algorithm structure and other additional arithmetic operations.

The metaprogram begins and generates a sequence of butterfly operations before the target code is executed. Previously, it was composed of seven consecutive phases: creating a model of butterfly operations for time window *m* = 1; creating a model of butterfly operations for time window *m+oc* (feedforward technique), where *oc* is the overlap coefficient; preparation of RADIX-2 decimation indices; identification of duplicated operations; bit-reversal permutation indices preparation and finally target code generation. The basis of the solution is to generate a model of butterfly operations for time window *m* = 1 and the same for time window *m >* 1 moved over the input array INPUT with the specified overlapping coefficient (*oc*). Thanks to these models, RADIX-2 decimation is performed and duplicated butterfly operations are identified (widely described in [8]). Additionally, this step provides information on how large the auxiliary array should be for the feedforward technique. During runtime, while the FFT for time window *m* =1 is processed, the output from duplicated operations is moved to the auxiliary array. Then, for FFT and time window *m* = 2, data from the auxiliary array is copied into selected parts of the input array that allows to omit its recalculation [8]. A block diagram of the solution is presented in Figure 4.

The new metaprogram algorithm has been changed to adapt the Stockham solution. Bit-reversal permutation indices must still be prepared, but only to preserve information about the place where the final stage output will be written. There is no need to generate code for an additional phase of indices rearrangement in the target program. All procedures connected to this phase belong only to the metaprogram. Listings 2 and 3 and present the changes that were made in the metaprogram.

**Listing 2.** Metaprogram that presents a part of butterfly operations generation (BEFORE STOCKHAM ALG. ADJUSTMENT). The generated code consists of variables like: *tempR, tempI, FR, FI, FR2, FI2* (real and imaginary part) and tables: *current* and *transitiontable* for feedforward solution (presented with green color). Last loop stands for bit-reversal permutation. The array *operations_rest* stands for the sequence of butterfly operation parameters. Each operation has 5 elements (shown with red color)…

int[] operations_rest = operation_for_rest_stages; for (int i = 0; i < operations_rest.length; **i += 5**) { method_computeImpl_BUILDER.startBlock(); method_computeImpl_BUILDER.addStatement("tempR = current[$L]", operations_rest[i + 1]); method_computeImpl_BUILDER.addStatement("tempI = current[$L]", operations_rest[i + 1] + 1); optimizeTwiddleFactorMultiplication(method_computeImpl_BUILDER, "FR", "tempR", *FACTOR2*[operations_rest[i + 2]]); optimizeTwiddleFactorMultiplication(method_computeImpl_BUILDER, "FI", "tempR", *FACTOR3*[operations_rest[i + 2]]); optimizeTwiddleFactorMultiplication(method_computeImpl_BUILDER, "FR2", "tempI", *FACTOR3*[operations_rest[i + 2]]); optimizeTwiddleFactorMultiplication(method_computeImpl_BUILDER, "FI2", "tempI", *FACTOR2*[operations_rest[i + 2]]); if (*FACTOR3*[operations_rest[i + 2]] != 0) { method_computeImpl_BUILDER.addStatement("FR -= FR2"); } if (*FACTOR2*[operations_rest[i + 2]] != 0) { method_computeImpl_BUILDER.addStatement("FI += FI2"); } if (operations_rest[i + 3] >= 0) { method_computeImpl_BUILDER.addStatement("transitionTable[$L] = current[$L] = current[$L] - FR", operations_rest[i + 4], operations_rest[i + 1], operations_rest[i]); method_computeImpl_BUILDER.addStatement("transitionTable[$L] = current[$L] = current[$L] - FI", operations_rest[i + 4] + 1, operations_rest[i + 1] + 1, operations_rest[i] + 1); method_computeImpl_BUILDER.addStatement("transitionTable[$L] = current[$L] = current[$L] + FR", operations_rest[i + 3], operations_rest[i], operations_rest[i]); method_computeImpl_BUILDER.addStatement("transitionTable[$L] = current[$L] = current[$L] + FI", operations_rest[i + 3] + 1, operations_rest[i] + 1, operations_rest[i] + 1); } else { method_computeImpl_BUILDER.addStatement("current[$L] = current[$L] - FR", operations_rest[i + 1], operations_rest[i]); method_computeImpl_BUILDER.addStatement("current[$L] = current[$L] - FI", operations_rest[i + 1] + 1, operations_rest[i] + 1); method_computeImpl_BUILDER.addStatement("current[$L] = current[$L] + FR", operations_rest[i], operations_rest[i]); method_computeImpl_BUILDER.addStatement("current[$L] = current[$L] + FI", operations_rest[i] + 1, operations_rest[i] + 1); } }
*// bit reversal permutation* int[] operations_bitReverse = operation_for_bit_reverse; for (int i = 0; i < operations_bitReverse.length; i += 5) { method_computeImpl_BUILDER.startBlock(); method_computeImpl_BUILDER.addStatement("tempR = current[$L]", operations_bitReverse[i]); method_computeImpl_BUILDER.addStatement("tempI = current[$L]", operations_bitReverse[i] + 1); method_computeImpl_BUILDER.addStatement("current[$L] = current[$L]", operations_bitReverse[i], operations_bitReverse[i + 1]); method_computeImpl_BUILDER.addStatement("current[$L] = current[$L]", operations_bitReverse[i] + 1, operations_bitReverse[i + 1] + 1); method_computeImpl_BUILDER.addStatement("current[$L] = tempR", operations_bitReverse[i + 1]); method_computeImpl_BUILDER.addStatement("current[$L] = tempI", operations_bitReverse[i + 1] + 1); }

**Listing 3.** Metaprogram changes that stands for stockham’s idea adjustment. Comparing to Listing 2, there is an additional array called *stockhamtable* that is filled depends on FFT stage. Also there is no bit-reversal permutation generation loop.

**…** int[] operations_rest = operation_for_rest_stages; for (int i = 0; i < operations_rest.length; **i += 6**) { String tableSource = operations_rest[i + 5]==0 ? "stockhamTable":"current"; String tableTarget = operations_rest[i + 5]==1 ? "stockhamTable":"current"; method_computeImpl_BUILDER.startBlock(); method_computeImpl_BUILDER.addStatement("tempR = " + tableSource + "[$L]", operations_rest[i + 1]); method_computeImpl_BUILDER.addStatement("tempI = " + tableSource + "[$L]", operations_rest[i + 1] + 1); optimizeTwiddleFactorMultiplication(method_computeImpl_BUILDER, "FR", "tempR", *FACTOR2*[operations_rest[i + 2]]); optimizeTwiddleFactorMultiplication(method_computeImpl_BUILDER, "FI", "tempR", *FACTOR3*[operations_rest[i + 2]]); optimizeTwiddleFactorMultiplication(method_computeImpl_BUILDER, "FR2", "tempI", *FACTOR3*[operations_rest[i + 2]]); optimizeTwiddleFactorMultiplication(method_computeImpl_BUILDER, "FI2", "tempI", *FACTOR2*[operations_rest[i + 2]]); if (*FACTOR3*[operations_rest[i + 2]] != 0) { method_computeImpl_BUILDER.addStatement("FR -= FR2"); } if (*FACTOR2*[operations_rest[i + 2]] != 0) { method_computeImpl_BUILDER.addStatement("FI += FI2"); } if (operations_rest[i + 3] >= 0) { method_computeImpl_BUILDER.addStatement("transitionTable[$L] = "+tableTarget+"[$L] = "+tableSource+"[$L] - FR", operations_rest[i + 4],bitReversed(operation_for_bit_reverse, operations_rest[i + 1],operations_rest[i + 5]), operations_rest[i]); method_computeImpl_BUILDER.addStatement("transitionTable[$L] = "+tableTarget+"[$L] = "+tableSource+"[$L] - FI", operations_rest[i + 4] + 1,bitReversed(operation_for_bit_reverse, operations_rest[i + 1] + 1,operations_rest[i + 5]), operations_rest[i] + 1); method_computeImpl_BUILDER.addStatement("transitionTable[$L] = "+tableTarget+"[$L] = "+tableSource+"[$L] + FR", operations_rest[i + 3], bitReversed(operation_for_bit_reverse, operations_rest[i],operations_rest[i + 5]), operations_rest[i]); method_computeImpl_BUILDER.addStatement("transitionTable[$L] = "+tableTarget+"[$L] = "+tableSource+"[$L] + FI", operations_rest[i + 3] + 1, bitReversed(operation_for_bit_reverse, operations_rest[i] + 1,operations_rest[i + 5]), operations_rest[i] + 1); } else { method_computeImpl_BUILDER.addStatement(tableTarget+"[$L] = "+tableSource+"[$L] - FR", bitReversed(operation_for_bit_reverse, operations_rest[i + 1],operations_rest[i + 5]), operations_rest[i]); method_computeImpl_BUILDER.addStatement(tableTarget+"[$L] = "+tableSource+"[$L] - FI", bitReversed(operation_for_bit_reverse, operations_rest[i + 1] + 1,operations_rest[i + 5]), operations_rest[i] + 1); method_computeImpl_BUILDER.addStatement(tableTarget+"[$L] = "+tableSource+"[$L] + FR", bitReversed(operation_for_bit_reverse, operations_rest[i],operations_rest[i + 5]), operations_rest[i]); method_computeImpl_BUILDER.addStatement(tableTarget+"[$L] = "+tableSource+"[$L] + FI", bitReversed(operation_for_bit_reverse, operations_rest[i] + 1,operations_rest[i + 5]), operations_rest[i] + 1); } }

As it can be seen above (Listing 2, Listing 3) STFT code generator was changed. Listings show that the main loop that is used to prepare target program goes through all butterfly operations. Its configuration is stored in *operations_rest* array, and iterator increase by 5 for each operation. After change it is increased with 6 because additional parameter was introduced. This parameter handle information about the processed stage. Last stage is indicated by value 0, thus for *last stage −1* this parameter will be set to 1. If the last stage is detected, source table name for STFT operation is set to *stockhamtable*, otherwise it is set to *current*. Target table name depends on one before last stage. Thanks to this approach metaprogram generates the sequence of target application operations, where data is copied between current and auxiliary array as it was proposed by Stockham.

**Figure 5:**
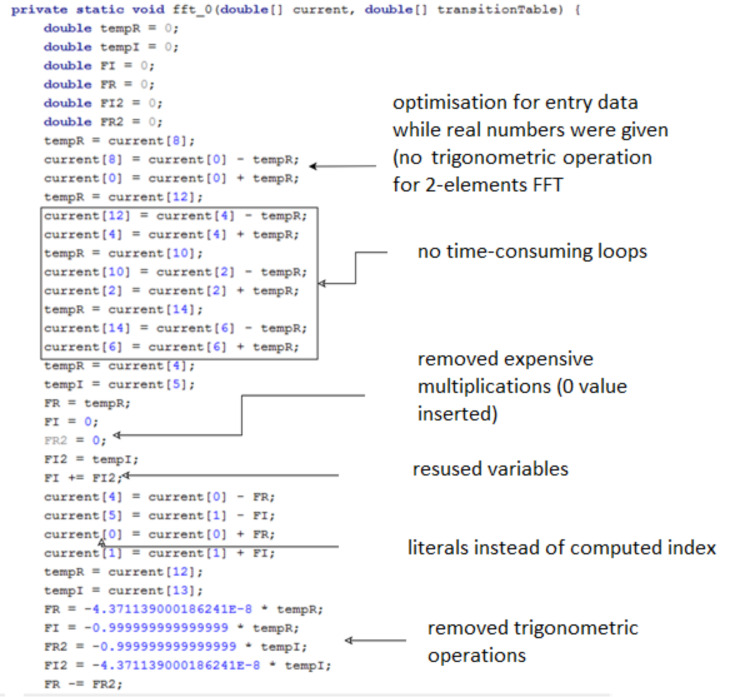
Profit from the application of the metaprogramming technique (generated code of the Short Time Fourier Transform algorithm).

The result of running the metaprogram is the optimized code of the target application. For the STFT algorithm, the code fragment is presented in Figure 5. Places in the resulting program were indicated that best represent the above-mentioned profit. This code was generated for 8 element FFT series. Main advantage of use metaprogram is to reduce trigonometric operations, omit indices computations and possibility to remove duplicated butterfly operations. The solution is not based on conditional operations, but completely eliminates redundant code in the target application.

## 3. Results

In order to examine the efficiency of the prepared solution, a personal computer equipped with an Intel Core i7-4710HQ processor and 12GB RAM was used. The underlying operating system was Windows 8. The metaprogram and target STFT code was implemented on a Java platform [Java(TM) SE Runtime Environment (build 1.8.0_231-b11)]. The whole verification process was supported by a JMH library created specifically for the purpose of performance analysis. Data that is processed is compliant with IEEE 754 double precision floating-point arithmetic.

Test procedure consisted of four steps: code preparation, test preparation, test execution, and results analysis. Code preparation means STFT code generation. This phase is not measured, because once the code has been generated the result may be saved in the repository. Next, test parameters are prepared. These consist of the window size (*N* = [8…2048]) and the number of windows for STFT (*m* = 200,000). Finally, an input array of real values is generated as common data for all the tested algorithms.

**Figure 6:**
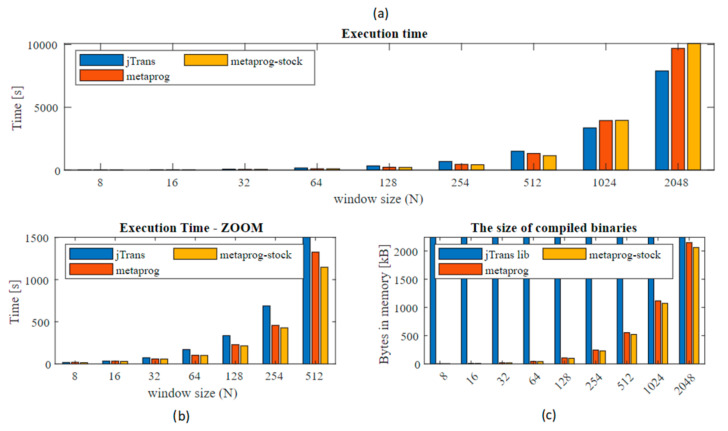
Comparison of execution time for jTransform library, metaprogram (metaprog), metaprogram with stockham extension (metaprog-stock). Analysis of compiled, generated binaries size. (**a**) execution times; (**b**) zoom for *N* = [8…512]. (**c**) The size of compiled, generated binaries for the analyzed algorithms.

**Figure 7:**
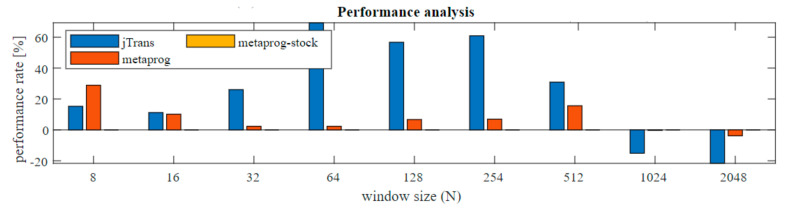
Performance improvement test results for STFT solutions (metaprog-stock as reference).

The measurement phase starts with a so-called warm-up. This approach is commonly used to eliminate the influence of Java virtual machine behavior connected with the just-in-time compilation facility. It was assumed and confirmed that 5 iterations are sufficient to omit this effect. The tested solutions were *jTransform* (DoubleFFT_1D.complexForward method) [38], metaprogramming STFT, and metaprogramming STFT with the Stockham extension. The results of execution time measurements are presented in Figure 6 and Figure 7. For each data input length (*N*) tests were performed 30 times. Final results presented below stands for the average values.

The execution times for the three solutions are very similar. Major changes are recorded above *N* ≥ 1024, where the jTrans algorithm is clearly better due to its possibility of concurrent computing, but when lower *N* are examined the metaprogramming solutions show higher performance (zoom). In order to better data presentation, the percentage changes were computed (Equation (2)) and presented in Figure 7. As the reference algorithm (*REF)* the metaprog-stock was used.

(2) $$P=\frac{\tau -\tau_{REF}}{\tau_{REF}}*100\%$$

Authors performed additional tests on other operating systems to exclude an influence of execution environment. Linux (Zorin distro) was used. Computer was equipped with 32 GB RAM and Intel^®^ Core™ i7 10gen 10750H processor. Results were different but ratio between three solutions is similar. Results were presented in Table 1.

**Figure 8:**
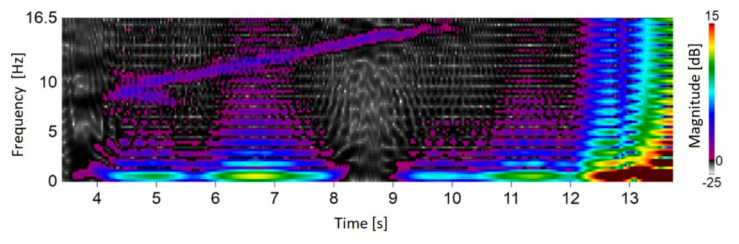
The result of STFT application for ECT data. Material concentration changes in silo cross-section during discharging process (own elaboration).

The test stand was prepared at the Laboratory of T. Dyakowski at the Institute of Applied Computer Science at the Lodz University of Technology. The elements of the structure and measurement system consisted of: silo model with a diameter of 200 mm and a height of 2000 mm (wall thickness 4 mm), 8-electrode capacitive sensor with a tomograph (30 frames/s). During the experiment, time courses of material concentration vibrations were obtained, which were processed using STFT. Further processing allowed to extract dominant harmonic which was changing during silo discharging. The experiments were carried out for non-cohesive and cohesive loose materials. Medium-grain quartz sand (medium grain diameter) was used as a non-cohesive material *d*_50_ = 0.8 × 10^−3^ m. A mixture of medium-grained sand and water was used as the cohesive material [1]. Result was presented in Figure 8. Figure presents material concentration changes in silo cross-section during discharging process. The rectangular window was used.

**Figure 9:**
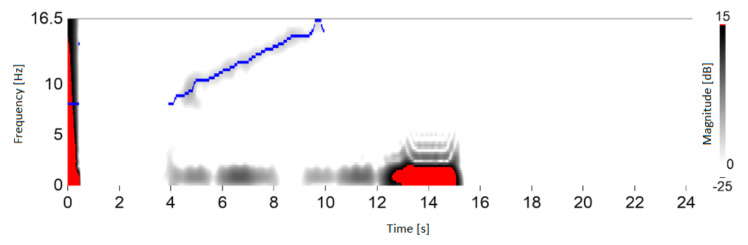
STFT application for ECT data. Dominant harmonic visualization for ECT data while discharging silo with grain (own elaboration).

Figure 8 presents that oscillation in range of 7–16 Hz was recorded. This experiment confirms the significance and value of the research conducted on the optimization of time-frequency transform algorithms for material conveying systems. Further process improvements involved dominant harmonic identification algorithms. Algorithm output presented in Figure 9.

## 4. Analysis and Discussion

The solution was implemented with JavaPoet library. Using this tool, both the metaprogram and the final application could be prepared in the same programming language, which has a significant impact on its maintenance. Programmer is not forced to switch between different programming languages. JavaPoet is a sufficient tool to keep target application syntax valid. Even the output code is very extensive. Each line meets Java syntax requirements and will not cause compilation errors.

The improvement achieves 60% acceleration in the range mainly for *N* = 64–256, compared to other common solutions such as the JTransform library. The improvement was also recorded comparing current solution to the previous one. Difference is around 2.3% to 28% compared to the former solution without the Stockham extension.

Very interesting is fact that for small *N* (8, 16) and large (1024 and more) the presented solution performance is slower than the reference (jTransform). This observation I would divide into two separated problems. First refers to *N* = 8, 16. As it can be seen in Figure 7 jTransform is better than metaprogram solution but worse than metaprogram with stockham extension. Metaprogram solution had bit reverse permutation for *N* = 8 and when it was removed then the code sped up its performance. JTransform for *N* = 4 and *N* = 8 doesn’t have reordering phase. For *N* > 32 there is a function ‘bitrv2conj’ which seems to be very expensive. Second issue: This results from the fact that jTransform solution uses strategy pattern and adjust implementation based on input array size *N*. When *N* is larger than allowed array size (hardcoded: N*2 > 1073741824) then so called large array strategy is performed. If *N* is not a power of two then Bluestein plan or mixed_radix plan is triggered otherwise split_radix is used. In initialization mode specific preprocessing is done. For an example the lookup table with trigonometric values is also generated. It was possible to retrieve the source code from repository, but despite this, due to the complexity, the algorithm is difficult to read. For sure algorithm is based on the parallel computing. It uses *ConcurrencyUtils.submit()* method to trigger parallel computations that extremely speed up solution for higher *N*.

Despite of execution time analysis, the size of generated application was also studied. As can be seen in Figure 8, the presented solution has some disadvantages. Code size grows rapidly with increasing window size and finally (for *N* = 2048) gives almost the same value as the whole jTransform library. For an instance, metaprog has 245 KB and metaprog-stock 229 KB for *N* = 256, and increasing to 554 KB and 522 KB for *N* = 512, respectively. Obviously, for modern computers these numbers are not crucial.

Memory complexity was not studied as it change only due to utilizing auxiliary table that is size of *N* (length of input array).

Generating the code of the Short Time Fourier Transform algorithm as presented in this article has many advantages:

- introduction to the solution the domain analysis of the problem; the principle of solving the problem remains unchanged, but it is possible to improve program efficiency,
- possibility to control the code that is generated,
- removal of loops (reduction of computation time),
- removal of calculations resulting from known parameters,
- control of table indexes during application generation,
- generator parameterization (parameter—time window length, parameter—FFT frame overlap coefficient),
- reduction of arithmetic operations on the basis of mathematical properties.

The results clearly show that it is beneficial to use the new tool for STFT when the window size is in the range of *N* = [8–512]. Despite its restrictions, the solution is sufficient for short time windows data processing, especially when the non-stationary signal changes rapidly and the time window should be limited. An example of its possible application may be the time-frequency analysis of the Electrical Capacitance Tomography data (ECT). ECT is used to measure the distribution of material concentration in a silo cross-section during the discharging process. The measurement frequency is low and reaches 30–150 Hz [1].

## 5. Conclusions

It is extremely difficult to improve such a common signal processing algorithm as STFT. However, this paper has presented a consecutive solution that allows to further reduce its execution time.

The method was elaborated as an extension of a previously developed application, in order to improve its efficiency. Adaptation of the Stockham concept was crucial to omit bit-reversal output data modification and obtain STFT values in order.

This was possible at the expense of memory needed to transfer intermediate results between the final two stages. The increased memory requirements are negligible due to the current capabilities of modern computers. Thanks to use of the metaprogramming technique, it was possible to move time-consuming operations into the code generation phase. The improvement achieves 60% acceleration.

It is worth to emphasize that the same as jTransform the solution is characterized by high portability as it is written in Java language. Future work will explore the possibility of applying parallel computation approaches to metaprogramming, which can dramatically increase the performance of STFT computations, particularly for larger input data.

## Figures and Tables

**Table 1:** Execution times for three solutions and two runtime environments: Windows and Linux (values in ms).

	N								
Solution	8	16	32	64	128	256	512	1024	2048
jTrans _linux	6.72	13.29	40.96	86.04	186.81	384.91	808.04	1709.00	3849.00
metaprog _linux	7.57	14.07	30.29	65.71	149.44	321.32	716.68	1815.00	4618.00
metaprog_stock _linux	6.68	12.16	23.54	58.24	136.54	292.10	688.98	1844.00	4887.00
jTrans (windows)	16.89	33.45	72.47	170.33	335.31	688.22	1501.98	3357.00	7891.00
Metaprog (windows)	18.89	33.13	58.84	102.95	228.34	457.53	1326.59	3943.00	9686.00
metaprog_stock(windows)	14.65	30.08	57.49	100.61	213.99	427.76	1147.36	3956.00	10,072.00

## Data Availability

Not applicable.
